# Nanoparticles from Equine Fetal Bone Marrow-Derived Cells Enhance the Survival of Injured Chondrocytes

**DOI:** 10.3390/ani10101723

**Published:** 2020-09-23

**Authors:** Ki Hoon Kim, Tae Sub Park, Byung-Wook Cho, Tae Min Kim

**Affiliations:** 1 Graduate School of International Agricultural Technology, Seoul National University, Pyeongchang Daero 1447, Pyeongchang, Gangwon-do 25354, Korea; hoon2087@snu.ac.kr (K.H.K.); taesubpark@snu.ac.kr (T.S.P.); 2 Institutes of Green-Bio Science and Technology, Seoul National University, Pyeongchang Daero 1447, Pyeongchang, Gangwon-do 25354, Korea; 3 Department of Animal Science, College of Natural Resources and Life Sciences, Pusan National University, Samnangin-ro 1268-50, Miryang, Gyeongsangnam-do 50463, Korea; bwcho@pusan.ac.kr

**Keywords:** bone marrow cells, joint diseases, equine chondrocytes, nanoparticles

## Abstract

**Simple Summary:**

Reports on the potential of using mesenchymal stem cells (MSCs) for treating equine degenerative joint diseases have been increasing over the past few years, in spite of limitations such as uncontrolled differentiation, immunogenicity, and tumorigenicity. We elucidated an allogenic alternative, where equine bone marrow-derived cells (BMC)-derived nanoparticles (BMC-NPs) can be used to promote the growth of chondrocytes, and simultaneously reduce their cytokine-induced apoptosis. The role of BMC-NPs in activation of signaling pathway molecules was also established.

**Abstract:**

Recent studies have shown that mesenchymal stem cells (MSCs) can play a restorative role against degenerative joint diseases in horses. The purpose of this study was to investigate whether fetal bone marrow-derived cells (BMC)-derived nanoparticles (BMC-NPs) can stimulate the survival of equine chondrocytes. Equine fetal BMCs were isolated and characterized, and the role of BMC-NPs s in equine chondrocytes undergoing inflammatory cell death was examined. BMCs have several characteristics, such as the potential to differentiate into chondrocytes and osteocytes. Additionally, BMCs expressed immunoregulatory genes in response to treatment with tumor necrosis factor-alpha (TNF-α) and Interleukin 1 beta (IL-1β). We found that BMC-NPs were taken up by equine chondrocytes. Functionally, BMC-NPs promoted the growth of chondrocytes, and reduced apoptosis induced by inflammatory cytokines. Furthermore, we observed that BMC-NPs upregulated the phosphorylation of protein kinase B (Akt) in the presence of IL-1β, and reduced the phosphorylation of TNF-α-induced activation of extracellular signal-regulated kinase 1/2 (ERK1/2) in the chondrocytes. Cumulatively, our study demonstrated that equine fetal BMC-NPs have the potential to stimulate the survival of chondrocytes damaged by inflammatory cytokines. Thus, BMC-NPs may become an alternative cell-free allogenic therapeutic for degenerative joint diseases in horses.

## 1. Introduction

Joint disease is one of the most prevalent disorders in both companion and athletic horses, which occurs by continuous injuries in joint tissues including the cartilage, synovium, and subchondral bone [1]. Articular cartilage is a hyaline tissue that enables bones to contact and glide over, ultimately providing resilience, support, and movement with almost no friction [2]. Lameness caused by joint injuries can lead to reduced training days, decreased performance, and ultimately an early retirement. It was reported that a substantial number of thoroughbred horses that had been euthanized were diagnosed with arthritis in metacarpophalangeal joints [3]. Similarly, one prospective observational study revealed that the occurrence of injuries in metacarpo- and metatarso-phalangeal joints were an important cause of morbidity, based on the results of daily exercise performance and diagnosis [4].

Currently, several medications are available for joint diseases in equine, e.g., hyaluronic acid, chondroitin sulfate, nonsteroidal anti-inflammatory drugs (NSAIDs), and corticosteroids [5]. However, these methods mostly are effective for reducing the inflammatory symptom. Thus, other alternatives, i.e., cellular therapies using autologous or allogenic origins are now being developed, mostly due to their biological characteristics and therapeutic potential for tissue regeneration [6]. Recently, Luis et al. reported that repeated intra-articular injection of allogenic adipose-derived MSCs was able to reduce the lameness for 90 days, while reducing the administration of anti-inflammatory drugs [7]. Other study demonstrated that a combination of chondrogenic-induced MSCs and equine allogenic plasma led to an improvement of lameness score, lower glycosaminoglycan concentration and higher viscosity in synovial fluid in experimental groups [8]. The successes of MSC-based therapies in equine osteoarthritis (OA) was reported, however, several limitations such as uncontrolled differentiation potential, early cellular senescence in vivo, immunogenicity, and tumorigenicity, remains to be solved to make MSCs become safer and therapeutically feasible [9,10].

Extracellular vesicles (EVs) are cell-derived nanoparticles enclosed with lipid-bilayer membrane, and it is being recognized that they play multiple role in intercellular communication [11]. They contain various bioactive cargos such as various species of RNA (messenger RNA and small RNA), proteins, enzymes, lipids, and short DNA sequences [12,13]. Given that EVs contain bioactive molecules that can represent the physiological or molecular characteristics of the original cells, the potential of their use as an alternative way as cell-free therapeutics has been recently increased [14]. Not surprisingly, the therapeutic function of MSC-derived EVs on joint or cartilage diseases have been reported in various studies conducted in vitro and in vivo. Mechanistically, their therapeutic potentials have been reported to be mainly due to the ability to enhance the proliferation and attenuate the apoptosis of chondrocytes, and to modulate the immune reactivity [15,16]. For example, treatment of MSC-derived exosomes led to an activation of ERK and AKT signaling in cultured chondrocytes in vitro [17]. Also, infiltration of M2 over M1 macrophages was increased in the osteochondral tissue in a surgical defect created on the model [18]. In addition, EVs from mouse BM-MSCs was able to reduce the progression of osteoarthritis in a collagenase-induced arthritis model, as indicated by the reduction in osteoarthritis damage and apoptotic cells, together with a significant improvement of cartilage tissue regeneration. Additionally, it was shown that exosomal miR-92a-3p inhibited the expression of WNT5A expression, which led to a reduction in cartilage degradation [19]. Another study reported that EVs from mouse BM-MSCs inhibited the proliferation of T lymphocytes in a dose-dependent manner, and that contributed to formation of a fewer plasmablasts and more Breg-like cell in lymph nodes [20].

Despite the potential usefulness of cell-derived nanoparticles or EVs for cartilage regeneration, no study has conducted on their reparative role in equine chondrocytes. Herein, we isolated and investigated the basic characteristics of equine bone marrow-derived cells (BMCs) from fetal bone marrow. Subsequently, the stimulatory effect of BMC-derived nanoparticles (BMC-NPs) on the growth as well as the survival of apoptotic equine chondrocytes under inflammatory damage were demonstrated.

## 2. Materials and Methods

### 2.1. Isolation and Culture of Bone Marrow Cells (BMCs)

This study was approved by Institutional Animal Care and Use Committees of Seoul National University (SNU-171103-2), and was conducted in accordance with approved guidelines. The pregnancy of a 3-year-old Jeju mare was terminated on day 190 upon detecting several complications, including lack of fetal mobility, nearly absent fetal heartbeat, and severe uterine torsion. Briefly, the mare was administered xylazine (0.8 mg/kg, intravenously [iv]), followed by diazepam (0.04 mg/kg, iv), and ketamine (2.2 mg/kg, iv). After sedation, anesthesia was maintained with isoflurane (2.0–2.5%) in oxygen. The uterus was exteriorized using the ventral midline approach and the fetus was pulled out, followed by closure of the uterus and abdomen. The gross morphology of the fetus was normal, without any lesions of microbial infection or severe tissue necrosis. After both proximal and distal ends of the femurs were cut open, the BMCs were harvested through flushing with a 50 mL DMEM (Dulbecco’s modified eagle medium, Thermo Fisher Scientific, Waltham, MA, USA) supplemented with 10% FBS (Fetal bovine serum, Altas Biologicals, Fort Collins, CO, USA) and 1% Antibiotics-Antimycotics (Genedirex, Taoyuan, Taiwan) using a syringe attached with a 16-gauge needle. Harvested BMCs were cultured in MesenPRO™ RS (Thermo Fisher Scientific, Waltham, MA, USA) supplemented with 0.1% Mycozap (Lonza, Basel, Switzerland). Cells were replenished with fresh culture medium every four days. When the cell growth reached 90% confluence, BMCs were split into 1:4 by being treated with 0.05% Trypsin-EDTA (Genedirex, Taoyuan, Taiwan). The cells passaged 8–10 times were used in the experiments.

### 2.2. Culture of Equine Chondrocytes

Equine primary chondrocytes (purchased from Cellider biotech, Zaragoza, Spain) were cultured in DMEM (Thermo Fisher Scientific, Waltham, MA, USA) with 10% FBS with 1% Antibiotics-Antimycotics (Genedirex, Taoyuan, Taiwan) under 5% CO_2_ condition at 37 °C. When the cells reached 90% confluence, chondrocytes were split into 1:2 or 1:3 by being treated with 0.05% Trypsin-EDTA (Genedirex, Taoyuan, Taiwan).

### 2.3. Co-Culture of Equine Chondrocytes with BMCs

Chondrocytes and BMCs (3.0 × 10^5^ cells/well for both cells) were separately cultured overnight on the bottom and the upper insert, respectively, in a 6-well transwell system (SPLInsert™ Hanging, SPL Life Sciences, Pocheon-si, Korea). After overnight culture, upper insert (BMCs) were subsequently hanged over the well where chondrocytes are being cultured. Afterwards, chondrocytes were co-cultured with BMCs in serum-free DMEM (Thermo Fisher Scientific, Waltham, MA, USA) for designated periods. Chondrocytes cultured in DMEM supplemented with (2.0%) or without FBS were used as positive and negative controls, respectively. To measure the growth of chondrocytes, the cell viability was measured by using the CellTiter-Glo 3D cell viability assay kit (Promega, Madison, WI, USA). The intensity of luminescence by cellular ATP was measured by Cytation 5 (BioTek, Winooski, VT, USA).

### 2.4. Flow Cytometry

BMCs were trypsinized and washed twice before resuspension in PBS containing 4% FBS. All stainings were performed using 1 × 10^6^ in 100 μL. Cell suspensions were incubated at 4 °C for 30 min with antibodies (1:100) against mouse anti-human CD29 (clone TS2/16, BioLegend, San Diego, CA, USA), mouse anti-CD34 (clone 4H11, Invitrogen, Carlsbad, CA, USA), mouse anti-CD90 (clone MRC OX-7, Abcam, Cambridge, UK), mouse anti-human CD105 (clone SN6, Bio-rad, Hercules, CA, USA), and rat anti-CD44 (clone IM7, Invitrogen, Carlsbad, CA, USA). Goat anti-mouse IgG H&L Dylight 488 (Abcam, Cambridge, UK) and anti-rat IgG Alexa Fluor 488 (BioLegend, San Diego, CA, USA) were used for secondary antibodies. The reactivity was analyzed using BD FACS Canto™ II Cytometer and FACS DIVA software (Ver 6.1.3, BD Bioscience, Franklin Lakes, NJ, USA).

### 2.5. Cytokine Treatment in BMCs and qRT-PCR

BMCs (4 × 10^5^ cells in 6-well plate) were treated with TNF-α (10 ng/mL, Peprotech US, Cranbury, NJ, USA) or IL-1β (10 ng/mL, Peprotech US, Cranbury, NJ, USA) for 24 or 48 h, and then the total RNA was extracted by Trizol^®^ (Invitrogen, Carlsbad, CA, USA). The concentration of total RNA was measured using DeNovix DS-11 (DeNovix, Wilmington, DE, USA), and then RNA was reverse transcribed with cDNA Synthesis Kit (PhileKorea, Daejeon-si, Korea). qPCR was performed using the AccuPower^®^ 2X GreenStar qPCR Master Mix (Bioneer, Daejeon-si, Korea) in StepOne™ Real-Time PCR System (Applied Biosystems, Waltham, MA, USA). After the expression of each gene was normalized against Gapdh, the relative expression of each gene was calculate by the 2^−ΔΔCt^ method [21]. The sequences of primers are listed in Table S1.

### 2.6. In Vitro Differentiation of BMCs

BMCs were plated in triplicate in 4-well plate (SPL Life Sciences, Pocheon-si, Korea) for chondrogenic a pellet cultured incubated at 37 °C and 5% CO_2_. After one day, the culture medium was removed, and then replaced with StemPro chondrogenesis (Thermo Fisher Scientific, Waltham, MA, USA) medium. Osteogenic and Adipogenic differentiation were incubated at 37 °C and 5% CO_2_. After reaching 80% confluence, the culture medium was removed, and StemPro osteogenesis and StemPro adipogenesis (Thermo Fisher Scientific, Waltham, MA, USA) were added to the cultures. All differentiation medium was renewed every three days, and their differentiation potential was examined after 15 days of differentiation. Chondrogenic differentiation was examined by staining with Alcian Blue staining kit (Lifeline Cell Technology, Frederick, MD, USA) to identify sulfated proteoglycans deposits. Osteogenic differentiation was examined by staining with 2% Alizarin Red staining kit (Lifeline Cell Technology, Frederick, MD, USA) to identify calcium deposits. Adipogenic differentiation was examined by staining with Oil Red O staining (Sigma-Aldrich, St. Louis, MO, USA) to identify lipid droplets.

### 2.7. Establishment of In Vitro Model of Chondrocyte Injury

Equine chondrocytes were cultured in DMEM supplemented with 0.1% FBS for 24 h. Cells were then harvested and re-seeded with various concentration of TNF-α or IL-1β (0.1, 1, 10, 100, and 200 ng/mL, Peprotech US, Cranbury, NJ, USA) in FBS-free DMEM for 24, 48, or 72 h. The viability of cells were measured by using CCK-8 assay.

### 2.8. CCK-8 Assay

Other than the co-culture experiments between chondrocytes and BMCs (Section 2.3), all analysis of cell viability/proliferation was measured by Cell Counting Kit-8 (Dojindo Laboratories, Kumamoto, Japan). Cells were seeded into 96-well plate (SPL Life Sciences, Pocheon-si, Korea) at a density of 5.0 × 10^3^ cells per well with 100 μL medium and incubated at 37 °C. At the designated time point, 10 μL CCK-8 solutions were added. After further incubation for 3 h at 37 °C, the amount of formazan generated by cellular dehydrogenase activity was measured (450 nm) by a microplate reader (TECAN, Mannedorf, Switzerland).

### 2.9. Collection of NPs

EV-depleted FBS was prepared by ultracentrifuge at 40,000× *g* for 8 h at 4 °C. Upon reaching 80% confluency, the culture media from BMCs were replaced with fresh medium supplemented with EV-depleted FBS (2%) and the cells were subsequently cultured for an additional 48 h. After incubation, the BMC culture medium was harvested, centrifuged 2000× *g* for 20 min at 4 °C, and the supernatants were filtered through 0.2 μm pore filters to remove the particle larger than 200 nm. And then, the supernatants were concentrated using Vivaspin 20 (100,000 MWCO) (Sartorius, Gottingen, Germany). Next, NPs were isolated by using Exo2 D™ Kit (Exosome plus, Suwon-si, Korea). The mixture was mixed by rocking for 30 min at 4 °C, followed by centrifugation at 3000× *g* for 30 min at 4 °C. Pellet was then resuspended in EV-free PBS that had been filtered through a 20 micrometer-pose sized syringe filter. The protein concentration was measured by the Pierce™ BCA Protein Assay kit (Thermo Fisher, Waltham, MA, USA). The aliquots were then stored at −80 °C.

### 2.10. Cryo-TEM

An aliquot (4 μL) of BMC-NPs were applied to glow-discharged (Glow discharge system, PELCO EasiGlow™, TED PELLA, Redding, CA) carbon-coated grids (Quantifoil, R2/2, 200 mesh, EMS, Hatfield, PA, USA). After the grids were then blotted for 90 s at 4 with 100% humidity, the samples were plunge-frozen for vitrification (Vitrobot mark IV, FEI, Hillsboro, Oregon). Images were collected on TEM microscope (Talos L120C, FEI, Hillsboro, Oregon) at 120 kV.

### 2.11. NTA Assessment

NPs were suspended EV-free distilled water, and then their particle size and concentration were measured using NanoSight300 (Malvern Panalytical, Malvern, UK).

### 2.12. Apoptosis Assay

Chondrocytes (2 × 10^5^ cells in 6-well plate) were treated with TNF-α (200 ng/mL) or IL-1β (200 ng/mL) for 24 or 48 h in the presence or absence of BMC-NPs (100 μg/mL). The apoptosis was measured with the Annexin V-FITC Apoptosis Detection Kit I (BD Bioscience, Franklin Lakes, NJ, USA). Cells were trypsinized and washed twice with cold PBS and then resuspended 1 × 10^6^ cells in 500 μL of binding buffer. Five microliters of Annexin V-FITC and 5 μL of PI (Propidium Iodide, 1 mg/mL) were added into the cell resuspended for 30 min at room temperature in the dark. The cells were analyzed BD FACS Canto™ II Cytometer and FACS DIVA software (Ver 6.1.3, BD Bioscience, Franklin Lakes, NJ, USA).

### 2.13. Immunobolotting

NPs and cellular protein total protein concentration evaluated by Pierce^TM^ BCA Protein Assay Kit (Thermo Fisher). NP and cell lysates were loaded into each well and separated by sodium dodecyl sulfate-polyacrylamide gel electrophoresis (SDS-PAGE, 10%). The protein bands were transferred to nitrocellulose membranes after which were blocked in 5% skim milk or 5% BSA. Primary antibodies (dilution 1:1000) against CD 63 (Abcam, Cambridge, UK), HSP 70, phospho-AKT, AKT, phospho-ERK, ERK (Santa Cruz, Dallas, TX, USA), or β-Actin (Abcam, Cambridge, UK) were incubated with the membrane at 4 °C overnight. After being washed three times with TBST (TBS with 0.1% Tween20^®^), membranes were incubated with horseradish peroxidase-linked anti-mouse or anti-rabbit secondary antibody for 1 h at room temperature. After being washed three times with TBST for 15 min each, the reactivity was examined by an enhanced chemiluminescence kit (Thermo Fisher Scientific, Waltham, MA, USA). The image of the membrane was taken using UV or white light on a Davinci-K Gel Imaging System (Davinch-K, Seoul, Korea). The density of bands was quantified by Image J (Version 1.50, National Institutes of Health, Bethesda, MD, USA).

### 2.14. BMC-NPs Uptake Study

Equine chondrocytes were cultured for 2 days at a density of 1.0 × 10^4^ cells per well in 8-well coated cover slides (SPL Life Sciences, Pocheon-si, Korea). BMC-NPs were labeled with PKH26^®^ red fluorescent dye according to the manufacturer’s instruction (Sigma-Aldrich, St. Louis, MO, USA). Labeled NPs (10, 50, 100 μg/mL) were co-cultured with equine chondrocytes for 24 h. For counterstaining, the nuclei and F-actin were stained with 4′,6-diandino-2-phenylindole (DAPI) (Sigma-Aldrich, St. Louis, MO, USA) and phalloidin (Abcam, Cambridge, UK), respectively. Images were obtained using the Cytation 5 (Biotek, Winooski, VT, USA).

### 2.15. Statistical Analysis

All data were presented as mean ± S.D. of at least three replications. For pairwise comparison, Student’s t-test was used. For more than three groups, one-way analysis of variation (ANOVA) followed by Tukey’s multiple comparison tests were conducted. All analyses were conducted by using GraphPad Prism software (Ver. 5.0 GraphPad Software, San Diego, CA, USA). P values less than 0.05 were considered as significantly different.

## 3. Results

### 3.1. Characterization of BMCs

**Figure 1:**
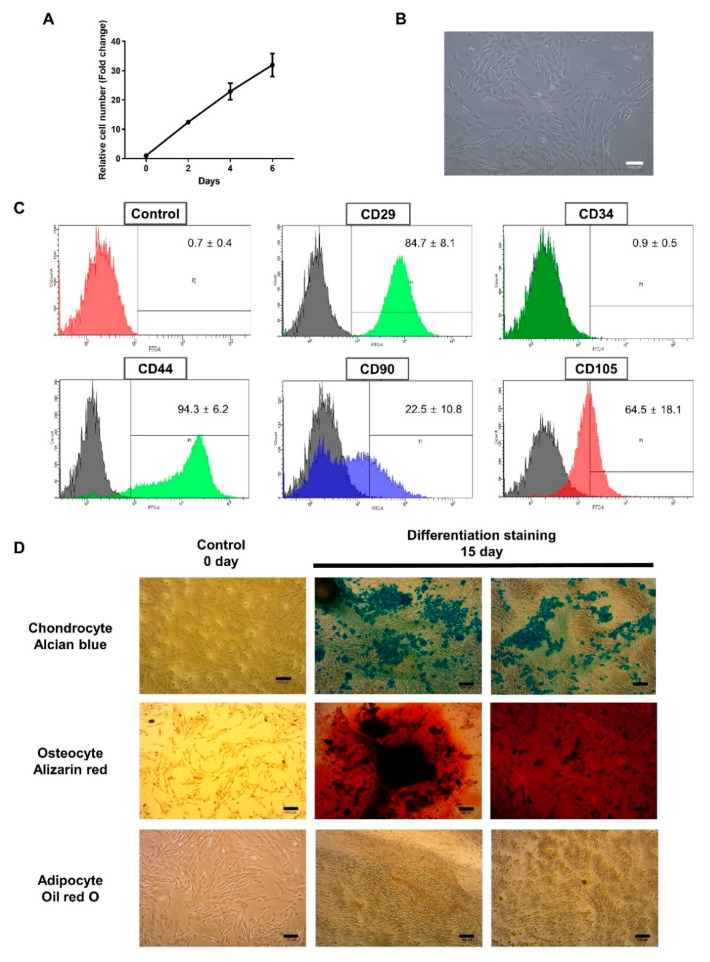
Characterization of equine fetal bone marrow-derived cells (BMCs). (**A**) Growth kinetics of BMCs (passages between 8–10). The fold change of the daily cell growth was obtained by normalizing against the initial number of cells at day 0. (**B**) The morphology of BMCs (passage 8). (**C**) Flow cytometry analysis of BMCs. (**D**) Microscopic images of differentiated cells from BMCs. BMCs were subjected to differentiation into chondrocytes, osteocytes, and adipocytes for fifteen days. Alcian blue, Alizarin red, and Oil red O staining were used for examining chondrogenic, osteogenic, and adipogenic differentiation, respectively. Scale bars are 100 μm.

First, the growth kinetics of BMCs was measured. As shown in Figure 1A, the number of BMCs were increased during a 6-day period (32 ± 6 folds). BMCs were observed spindle-like to fibroblastoid cell morphology (Figure 1B). Flow cytometric analysis of BMCs (at passage 8–10) showed that they are positive for several conventional MSCs surface markers such as CD44, CD90, and CD105, although the reactivity was heterogeneous among markers (Figure 1C) (84.7% for CD29, 22.5% for CD90, 64.5% for CD105, and 94.3% for CD44, respectively). A hematopoietic stem cell marker CD34 was negative. Then, BMCs were subjected to differentiation to chondrogenic, osteogenic, and adipogenic lineages for 15 days, and then stained for analyzing of the deposition of proteoglycan (Alcian blue), calcium (Alizarin Red), and neutral lipid (Oil Red O). As results, BMCs were readily differentiated to chondrogenic and osteogenic cells, however, their potential for adipogenic differentiation was low (Figure 1D). We also investigated whether the expression of immunoregulatory genes can be up-regulated in BMCs after being exposed to inflammatory stimuli. At 24 h, the expression of TGF-β1 and IDO was upregulated by either TNF-α or IL-1β, while no increase was detected in IL-10 message by TNF-α (Figure S1A). At 48 h, the expression of all genes was up-regulated after being treated with either TNF-α or IL-1β (Figure S1B).

### 3.2. Characterization of BMC-Derived NPs

**Figure 2:**
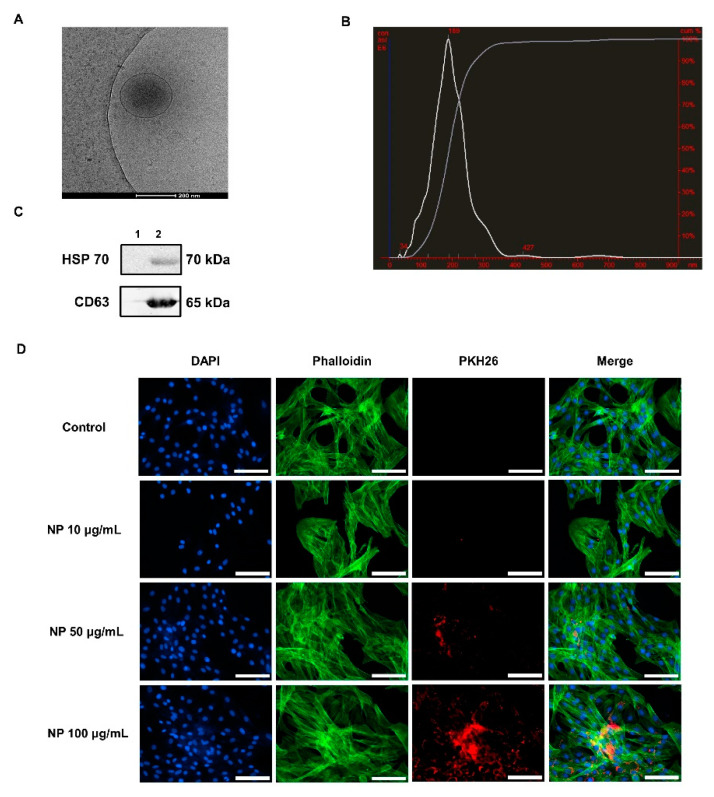
Characterization of (BMC)-derived nanoparticles (BMC-NPs). (**A**) The Cryo-EM image of BMC-NP. Scale bar is 200 nm. (**B**) NTA analysis of BMC-NPs. The mean diameter was 200 ± 73 nm. (**C**) Immunoblot analysis for HSP70 and CD63 in NPs. Lane 1 and 2 are negative control (blank lane) and BMC-NPs (5 μg of lysate), respectively. (**D**) Visualization of the uptake of BMC-NPs into equine chondrocyte. PKH26^®^ (red fluorescence)—stained BMC-NPs (10, 50, or 100 μg/mL) were incubated with equine chondrocyte for 24 h. Before analysis, cells were counterstained with Phalloidin 488 (green) and DAPI (blue). Scale bars are 100 μm.

NPs were isolated from the conditioned medium of BMCs after a 48 h period of culture. A brief process for isolating NPs was shown in Figure S2. First, the morphological characteristics of BMC-NPs were evaluated via cryo-TEM. As shown in Figure 2A, the nanoparticle had an intact and round shape (around 200 nm), with clear membranous structure. Subsequently, NTA (Nanoparticle Tracking Analyzer) analysis was conducted, and it was found that their mean diameter was 200 ± 73 nm (Figure 2B). Western blot analysis revealed that NPs were positive with tetraspanin CD63 and heat shock protein HSP70 (Figure 2C). To examine whether BMC-NPs can be taken up by equine chondrocytes, fluorescently labeled BMC-NPs were incubated with equine chondrocytes for 24 h, and their localization was analyzed. As shown in Figure 2D, the fluorescently-labeled spots were detected in the cytoplasm and peri-nuclear region of equine chondrocytes, but not in the nucleus, indicating that NPs were taken up into the equine chondrocytes. Collectively, these results suggest that BMC-NPs have a general characteristic of EVs, and they can be internalized into equine chondrocytes.

### 3.3. Assessment of the Role of BMCs and BMC-Derived NPs in the Viability of Equine Chondrocytes

**Figure 3:**
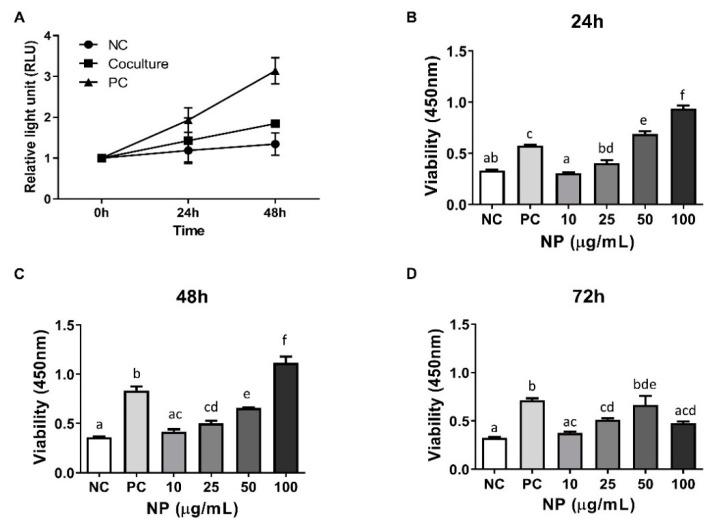
The effect of BMCs and BMC-derived NPs (BMC-NPs) on the proliferation of equine chondrocytes. (**A**) The growth profile of chondrocytes co-cultured with BMCs in serum-free condition. NC (negative control) and PC (positive control) indicate chondrocytes cultured without or with FBS (2%), respectively, in the absence of BMC. (**B**–**D**) The effect of BMC-NPs on the proliferation of chondrocytes. NC (negative control) and PC (positive control) indicate chondrocytes cultured without or with FBS (2%), respectively. FBS, Fetal bovine serum. All data are expressed mean ± SD from three replications. Bars labeled with different letters are significantly different (^a–f^ *p* < 0.05).

For functional analysis, the role of BMCs or BMC-NPs on the proliferation of equine chondrocytes was investigated. As results, the trophic effect of MSCs on the growth of chondrocytes was noted (Figure 3A) in serum-free culture condition, although the increase was lower than positive control (i.e., DMEM with 2% FBS in the absence of BMCs). Next, equine chondrocytes were treated with various concentration of BMC-NPs (10, 25, 50 and 100 μg/mL), and the increase of cells was analyzed after 24, 48, and 72 h of treatment. As shown in Figure 3B,C, BMC-NPs were able to stimulate the proliferation of chondrocytes in a time- and dose-dependent manner. Of note, the viability of equine chondrocytes at 72 h of treatment was less in 100 μg/mL compared with those treated with 50 μg/mL.

### 3.4. Establishing a Inflammatory Injury Model in Equine Chondrocytes

**Figure 4:**
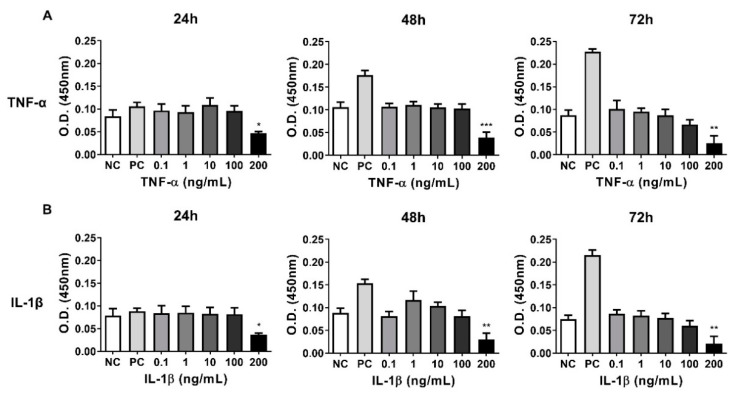
The effect of inflammatory cytokines on the viability of equine chondrocytes. The viability of equine chondrocytes incubated for 24, 48, and 72 h with different cytokines with TNF-α and IL-1β was measured. (**A**) Relative viability of chondrocytes after treated with TNF-α for 24, 48, and 72 h. (**B**) Relative viability of chondrocytes after treated with IL-1β in equine chondrocyte for 24, 48, and 72 h. NC (negative control) and PC (positive control) indicate chondrocytes cultured without or with FBS (2%), respectively. TNF-α, Tumor necrosis factor-α; IL-1β, Interleukin-1β; FBS, fetal bovine serum. All data are expressed as mean ± SD from three replications. * *p* < 0.05, ** *p* < 0.01, and *** *p* < 0.005 against negative control (NC).

Since no data was available on the effect of inflammatory stimuli on equine chondrocytes, a pilot experiment was conducted on the relationship between the viability and various concentration of TNF-α or IL-1β. Thus, the viability of equine chondrocytes was analyzed following treatment with various concentrations of cytokines (0.1, 1, 10, 100, and 200 ng/mL) for 24, 48, and 72 h. The results of CCK-8 assay showed that detectable death of chondrocytes was observed at 200 ng/mL (Figure 4).

### 3.5. The Effect of the BMC-NPs on the Survival of Injured Chondrocytes

**Figure 5:**
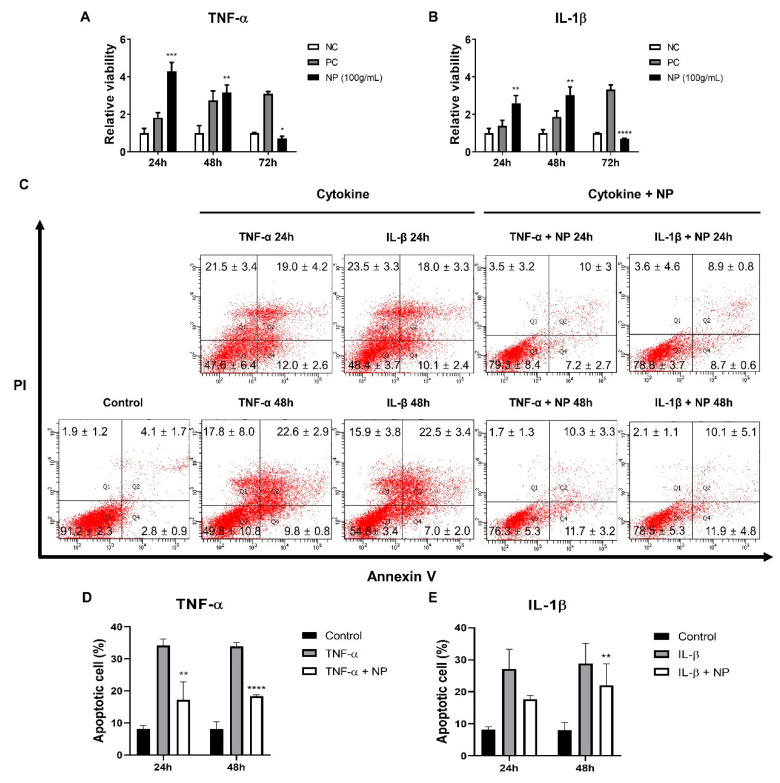
The effect of BMC-derived NPs on the survival of chondrocytes injured by inflammatory cytokines. (**A**,**B**) The survival of BMC-NPs on chondrocytes injured by TNF-α (**A**) or IL-1β (**B**) was examined at 24, 48, or 72 h. NC and PC are equine chondrocytes cultured without or with FBS (2%), respectively. (**C**) Representative image of the flow cytometry data on the apoptotic chondrocytes treated with or without BMC-NPs under inflammatory injury. Cells were stained with Annexin V-FITC/PI to determine the apoptotic cell death. (**D**,**E**) Comparison of the percentages of TNF-α- or IL-1β-induced apoptotic chondrocytes co-treated with or without BMC-NPs. All data are expressed mean ± SD from three replications. * *p* < 0.05, ** *p* < 0.01, *** *p* < 0.005, and **** *p* < 0.001 against negative control (**A**,**B**) or cells treated solely with TNF-α (**D**) or IL-1β (**E**).

This study was conducted to investigate whether BMC-NPs can exert their trophic function on chondrocytes that are under inflammatory injury. Based on the results from Figure 3, 100 μg/mL of BMP-NP were used. BMP-NP treatment led to enhanced viability that was even higher than the positive control. Such effect was not found at 72 h (Figure 5A,B). To further examine their role in enhancing the survival of injured chondrocytes, the apoptotic profile of chondrocytes was analyzed. As showed by Annexin V/PI staining, cytokine treatment led to a significant increase in the rate of apoptosis. In contrast, the apoptotic cell death induced by TNF-α was significantly reduced in cells co-treated with BMC-NPs at both 24 and 48 h, while such effect was found only at 48 h in IL-1β-induced cell death (Figure 5C–E).

### 3.6. Immunoblotting of Signaling Pathway Molecules

To determine the mechanism of the BMC-derived NPs on the survival of chondrocytes, the phosphorylation of AKT, which is known to be essential for cell proliferation [17], was examined. Also, the activity of ERK1/2 was also investigated, because they are known to play a critical role in inflammatory responses via changing the transcription of several genes [22,23,24]. After the equine chondrocytes were treated with TNFα or IL-1β, in the presence or absence with BMC-NPs, immunoblot analysis was conducted for the detection of AKT and ERK.

**Figure 6:**
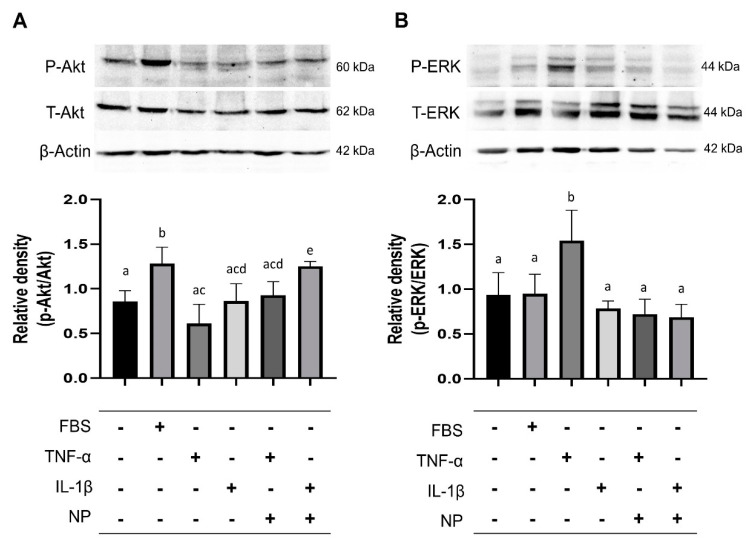
Immunoblot analysis of the signaling pathway molecules in chondrocytes. (**A**) Chondrocytes were stimulated with TNF-α or IL-1β for 24 h in the presence or absence of BMC-NPs, after which the cell extracts were analyzed by immunoblotting for AKT1 and ERK1/2. Beta-actin was used as the loading control. (**B**) Densitometric analysis of the relative level of phosphorylated AKT1 and ERK1/2. The value was normalized against that observed for negative control (NC). ERK, Extracellular signal-regulated kinase; FBS, Fetal bovine serum; TNF-α, Tumor necrosis factor-α; IL-1β, Interleukin-1β; NP, Nanoparticle. All data are expressed as mean ± SD from four replicates. Bars labeled with different letters are significantly different (^a–e^ *p* < 0.05).

Under inflammation induced by IL-1β, BMC-NPs promoted the activation of AKT (Figure 6A). Next, we found that ERK was increased by TNF-α, while co-treatment with BMC-NPs led to a marked reduction (Figure 6B).

## 4. Discussion

During last several decades, the reparative role of MSCs in joint diseases has been evidenced by various preclinical studies [25,26]. However, the possibility of immune rejection from allogenic origin, as well as the risk of tumor generation, can become a significant concern, which necessitates the development of other strategy equipped with less safety issue [27]. Also, difficulties in long-term storage and maintenance, while sustaining its consistency and viability, should be overcome to make these cells feasible for use in veterinary practice [28]. Initially, studies reported that MSCs take part in cartilage repair by the unique ability of MSCs to differentiate into several mesenchymal lineages, i.e., chondrocytes, which may replace the dead or injured chondrocytes [29]. However, it is now becoming accepted that the secretome of MSCs also plays a vital role in the repair of injured chondrocytes [30,31]. Since then, EVs including exosomes or microvesicles, which occupies a certain part of secretome, were found to be efficacious against cartilage regeneration. In particular, MSC-EVs have been effective in cartilage regeneration in a rodent model of osteoarthritis [18,20,32,33,34,35,36,37,38]. Despite such success and potential uses of MSC-EVs, no attempt was made on the use of MSC-derived nanoparticles or in cartilage repair in equine species. In this study, we investigated whether BMC-NPs have potential to stimulate the growth of equine chondrocytes, and found that BMC-NPs were readily taken up by equine chondrocytes, and that they promoted the proliferation of chondrocytes. Further, we found that BMC-NPs were able to rescue the apoptotic death of chondrocytes. Finally, we demonstrated that BMC-NPs led to an increased level of phosphorylated AKT as well as a reduction of ERK1/2 activation in equine chondrocytes that are under inflammatory stimuli.

Studies found that cellular characteristics of MSCs from fetal and adult tissues differ, and several advantages can be obtained from MSCS from fetal origin. It was reported that fetal MSCs have an increased proliferative activity [39,40,41], and an better therapeutic effects such as enhanced anti-inflammatory capacity, fitness, and homing ability compared with those obtained from adults [39,41,42]. It was also reported that in human, the ratio of the number of MSCs in newborns and aged adults are one out of a thousand and two million, respectively [43]. Thus, MSCs of fetal origin are found at a higher frequency in tissues than in adult MSCs, and are readily available in fetal and extra fetal tissues such as fetal liver, umbilical cord, umbilical cord blood, placenta, and amniotic fluid [39,44,45]. In addition, studies showed that fetal MSCs are more effective in avoiding immune recognition than adult MSCs [46,47]. Specifically, fetal MSCs are known to be less immunogenic than adult MSCs, because fetal MSCs have a lower surface of HLA Class I [41,46,48], and they do not express HLA class II on their surface. Lastly, fetal MSCs can be more suitable for obtaining specific mesenchymal lineages, e.g., due to an increased osteogenic potential compared with adult MSCs [49]. Indeed, a comparative study showed that many osteogenic genes were more abundantly expressed fetal than adult MSCs [50]. The differences in the potential of growth, differentiation, and immunoregulation between adult bone marrow-derived MSCs and fetal MSCs or BMCs remain unexplored in horses, thus further side-by-side comparison between two cell types is needed.

Signals downstream of stem cell-derived exosomes are often mediated via AKT or ERK pathways in various cells including chondrocytes [17,51,52,53]. For example, recent study demonstrated that exosomes from human embryonic stem cell-derived MSCs were able to facilitate the repair of osteochondral defect by 1) stimulating the proliferation by activating AKT signaling, 2) decreasing the apoptotic death of chondrocytes in the chondral tissue as well as IL-1β and TNF-α in synovial fluid in rats [18]. We found that BMC-NPs induced an increase of AKT signaling, while reduced the activity of ERK in the presence of IL-1B and TNF-α, respectively. These results suggest that BMC-NPs can promote the survival of chondrocytes via AKT, and that they also can inhibit the TNF-α/ERK1/2 inflammatory pathway. Further studies are warranted to clearly delineate the mechanisms how BMC-NPs can reduce the cell death induced by inflammatory cytokines in equine chondrocytes.

Studies have shown a varying degree of cell surface marker expression in horse MSCs. It was reported that the reactivity of CD90 was 67.7% in umbilical cord-derived-MSCs [54], while more heterogeneous findings are available regarding the population of CD105-postivie cells among study groups; Barberini et al. showed that adult MSCs from bone marrow, adipose tissue, and umbilical cord tissue was positive for CD105 [54], while other group reported that CD105 was not detectable in MSCs from bone marrow and adipose tissue [55]. Other study showed that adipose tissue-derived CD105^+^ MSCs were within the range of 21 to 74.4%, depending on the tissue sources (subcutaneous and intraperitoneal tissues) and passages [7]. Meanwhile, our results on the expression of CD29 and CD44 was comparable against other studies [55,56]. An extensive, comparative study also showed that the expression of CD29, CD90, and CD105 was 48.4, 11.4, and 35%, respectively, in adipose tissue derived MSCs, while 52.9, 0.3, 33.7%, respectively, in tendon-tissue derived MSCs [57]. Not surprisingly, these results demonstrate that immunophenotyping results differ significantly among investigations, indicating that various factors including cell sources and protocols for isolation and culture may account for these differences. Also, it should be considered that a more detailed studies for determining the specificity of common epitope and responding antibody is needed because the antibodies that work efficiently on equine species are not always commercially available.

Although the guidelines and general consensus are being renewed by scientific communities, the protocol for isolating, characterizing, and functional testing of EVs still remains heterogeneous among study groups. In equine species, few reports are available on the identification of MSC-derived EVs [58,59,60]. Mostly, EVs were collected by ultracentrifugation of the culture supernatant obtained during adipose tissue-derived MSCs (Ad-MSCs) culture. An early study showed that membrane vesicles from equine Ad-MSCs, as shown by scanning electron microscopy, had ability to stimulate the growth of endothelial cells [59]. Regarding the EV markers, it was recently shown that equine Ad-MSC-derived EVs were positive with CD63, CD9, and CD90 [60], while another study demonstrated the expression of CD90 and Flotillin-1, although the degree of reactivity was not clearly shown [58]. BMC-NPs from our study were positive with CD63 and HSP70, which are also markers used for exosome characterization [61,62,63,64]. As for the purification protocol, Klymiuk et al. reported that ultrafiltration method is superior to other methods (ultracentrifugation or precipitation) for isolating equine ADSC-exosomes in terms of the total yield as well as the aggregation issue during resuspension, although the biological functions of EVs from each preparation were not tested. The particle diameter was 178.7 ± 62.3 and 116.2 ± 38.3 nm in equine ADSC-exosomes isolated by ultracentrifugation and ultrafiltration, respectively [60], creating a significant difference on their diameter depending on the isolation methods. The method used in our present study is based on the aqueous two-phase system (ATPS), which is based on the incompatibility-based separation between the phases of two polymeric molecules, polyethylene glycol (PEG) and dextran [65,66]. Our data on the diameter of BMC-NPs was slightly larger than the previous study [60], indicating different physical characteristics among investigations. Since the isolation protocol can often create biological differences, standardization and further optimization is needed to improve the yield and to minimize the changes among batches.

Also, determining the optimal regime of nanoparticle treatment, e.g., concentration, frequency, and total dosage, is a critical not only for maximizing the therapeutic effect but also for reducing the toxicity. We noticed that higher concentration (100 μg/mL) of BMC-NPs led to a reduced cell survival compared with those treated with 50 μg/mL at 72 h. Given that the effects of BMC-NPs on injured chondrocytes were examined in the absence of other survival factors (e.g., FBS), the reduced survivability at high BMC-NP concentration may be related with toxic effect of lipid components (e.g., ceramide) that is derived from biogenesis processes [67]. Thus, it is suggested that further optimization is needed to minimize the toxic effect that can be possibly caused by a higher dosage of BMC-NPs.

Together, our results demonstrate that NPs from equine fetal bone marrow-derived cells can simulate the growth as well as the survival of apoptotic chondrocytes under inflammatory stimuli. The therapeutic effect of equine fetal BMC-NPs may be utilized to develop a novel strategy for treating joint disease in horses.

## 5. Conclusions

Our results demonstrate that nanoparticles secreted from equine fetal bone marrow-derived cells (BMC-NPs) can simulate the survival of apoptotic chondrocytes injured by inflammatory stimuli. The therapeutic effect of equine fetal BMC-NPs may be utilized to develop a novel strategy for treating joint disease in horses.

## Supplementary Materials

The following are available online at https://www.mdpi.com/2076-2615/10/10/1723/s1, Figure S1: The mRNA expression analysis of immunoregulatory genes in BMCs. Figure S2: The schematic workflow of isolation of BMC-NPs. Table S1: The primer sequences for qRT-PCR.

## References

[1] Ekstrand C., Bondesson U., Giving E., Hedeland M., Ingvast-Larsson C., Jacobsen S., Lofgren M., Moen L., Rhodin M., Saetra T. (2019). Disposition and effect of intra-articularly administered dexamethasone on lipopolysaccharide induced equine synovitis. Acta Vet. Scand..

[2] Hardingham T.E. (2010). Fell-Muir lecture: Cartilage 2010—the known unknowns. Int. J. Exp. Pathol..

[3] Neundorf R.H., Lowerison M.B., Cruz A.M., Thomason J.J., McEwen B.J., Hurtig M.B. (2010). Determination of the prevalence and severity of metacarpophalangeal joint osteoarthritis in Thoroughbred racehorses via quantitative macroscopic evaluation. Am. J. Vet. Res..

[4] Reed S.R., Jackson B.F., Mc Ilwraith C.W., Wright I.M., Pilsworth R., Knapp S., Wood J.L., Price J.S., Verheyen K.L. (2012). Descriptive epidemiology of joint injuries in Thoroughbred racehorses in training. Equine Vet. J..

[5] Broeckx S.Y., Seys B., Suls M., Vandenberghe A., Marien T., Adriaensen E., Declercq J., Van Hecke L., Braun G., Hellmann K. (2019). Equine Allogeneic Chondrogenic Induced Mesenchymal Stem Cells Are an Effective Treatment for Degenerative Joint Disease in Horses. Stem Cells Dev..

[6] Colbath A.C., Dow S.W., McIlwraith C.W., Goodrich L.R. (2020). Mesenchymal stem cells for treatment of musculoskeletal disease in horses: Relative merits of allogeneic versus autologous stem cells. Equine Vet. J..

[7] Marinas-Pardo L., Garcia-Castro J., Rodriguez-Hurtado I., Rodriguez-Garcia M.I., Nunez-Naveira L., Hermida-Prieto M. (2018). Allogeneic Adipose-Derived Mesenchymal Stem Cells (Horse Allo 20) for the Treatment of Osteoarthritis-Associated Lameness in Horses: Characterization, Safety, and Efficacy of Intra-Articular Treatment. Stem Cells Dev..

[8] Broeckx S.Y., Martens A.M., Bertone A.L., Van Brantegem L., Duchateau L., Van Hecke L., Dumoulin M., Oosterlinck M., Chiers K., Hussein H. (2019). The use of equine chondrogenic-induced mesenchymal stem cells as a treatment for osteoarthritis: A randomised, double-blinded, placebo-controlled proof-of-concept study. Equine Vet. J..

[9] Ardanaz N., Vazquez F.J., Romero A., Remacha A.R., Barrachina L., Sanz A., Ranera B., Vitoria A., Albareda J., Prades M. (2016). Inflammatory response to the administration of mesenchymal stem cells in an equine experimental model: Effect of autologous, and single and repeat doses of pooled allogeneic cells in healthy joints. BMC Vet. Res..

[10] Zayed M., Adair S., Ursini T., Schumacher J., Misk N., Dhar M. (2018). Concepts and challenges in the use of mesenchymal stem cells as a treatment for cartilage damage in the horse. Res. Vet. Sci..

[11] Kourembanas S. (2015). Exosomes: Vehicles of intercellular signaling, biomarkers, and vectors of cell therapy. Annu. Rev. Physiol..

[12] Murphy C., Withrow J., Hunter M., Liu Y., Tang Y.L., Fulzele S., Hamrick M.W. (2018). Emerging role of extracellular vesicles in musculoskeletal diseases. Mol. Asp. Med..

[13] Burke J., Hunter M., Kolhe R., Isales C., Hamrick M., Fulzele S. (2016). Therapeutic potential of mesenchymal stem cell based therapy for osteoarthritis. Clin. Transl. Med..

[14] Joo H.S., Suh J.H., Lee H.J., Bang E.S., Lee J.M. (2020). Current Knowledge and Future Perspectives on Mesenchymal Stem Cell-Derived Exosomes as a New Therapeutic Agent. Int. J. Mol. Sci..

[15] Li J.J., Hosseini-Beheshti E., Grau G.E., Zreiqat H., Little C.B. (2019). Stem Cell-Derived Extracellular Vesicles for Treating Joint Injury and Osteoarthritis. Nanomaterials (Basel).

[16] Liu Y., Lin L., Zou R., Wen C., Wang Z., Lin F. (2018). MSC-derived exosomes promote proliferation and inhibit apoptosis of chondrocytes via lncRNA-KLF3-AS1/miR-206/GIT1 axis in osteoarthritis. Cell Cycle.

[17] Zhang S., Teo K.Y.W., Chuah S.J., Lai R.C., Lim S.K., Toh W.S. (2019). MSC exosomes alleviate temporomandibular joint osteoarthritis by attenuating inflammation and restoring matrix homeostasis. Biomaterials.

[18] Zhang S., Chuah S.J., Lai R.C., Hui J.H.P., Lim S.K., Toh W.S. (2018). MSC exosomes mediate cartilage repair by enhancing proliferation, attenuating apoptosis and modulating immune reactivity. Biomaterials.

[19] Mao G., Zhang Z., Hu S., Zhang Z., Chang Z., Huang Z., Liao W., Kang Y. (2018). Exosomes derived from miR-92a-3p-overexpressing human mesenchymal stem cells enhance chondrogenesis and suppress cartilage degradation via targeting WNT5A. Stem Cell Res..

[20] Cosenza S., Toupet K., Maumus M., Luz-Crawford P., Blanc-Brude O., Jorgensen C., Noel D. (2018). Mesenchymal stem cells-derived exosomes are more immunosuppressive than microparticles in inflammatory arthritis. Theranostics.

[21] Livak K.J., Schmittgen T.D. (2001). Analysis of relative gene expression data using real-time quantitative PCR and the 2(-Delta Delta C(T)) Method. Methods.

[22] Xie L., Xie H., Chen C., Tao Z., Zhang C., Cai L. (2019). Inhibiting the PI3K/AKT/NF-κB signal pathway with nobiletin for attenuating the development of osteoarthritis: In vitro and in vivo studies. Food Funct..

[23] Viatour P., Merville M.P., Bours V., Chariot A. (2005). Phosphorylation of NF-kappaB and IkappaB proteins: Implications in cancer and inflammation. Trends Biochem. Sci..

[24] Park M.H., Hong J.T. (2016). Roles of NF-kappaB in Cancer and Inflammatory Diseases and Their Therapeutic Approaches. Cells.

[25] Nejadnik H., Hui J.H., Feng Choong E.P., Tai B.-C., Lee E.H. (2010). Autologous bone marrow–derived mesenchymal stem cells versus autologous chondrocyte implantation: An observational cohort study. Am. J. Sports Med..

[26] Lim C.T., Ren X., Afizah M.H., Tarigan-Panjaitan S., Yang Z., Wu Y., Chian K.S., Mikos A.G., Hui J.H. (2013). Repair of osteochondral defects with rehydrated freeze-dried oligo[poly(ethylene glycol) fumarate] hydrogels seeded with bone marrow mesenchymal stem cells in a porcine model. Tissue Eng. Part A.

[27] Bjorge I.M., Kim S.Y., Mano J.F., Kalionis B., Chrzanowski W. (2017). Extracellular vesicles, exosomes and shedding vesicles in regenerative medicine—A new paradigm for tissue repair. Biomater. Sci..

[28] Toh W.S., Lai R.C., Hui J.H.P., Lim S.K. (2017). MSC exosome as a cell-free MSC therapy for cartilage regeneration: Implications for osteoarthritis treatment. Semin. Cell Dev. Biol..

[29] Chen K., Man C., Zhang B., Hu J., Zhu S.S. (2013). Effect of in vitro chondrogenic differentiation of autologous mesenchymal stem cells on cartilage and subchondral cancellous bone repair in osteoarthritis of temporomandibular joint. Int. J. Oral Maxillofac. Surg..

[30] Toh W.S., Foldager C.B., Pei M., Hui J.H. (2014). Advances in mesenchymal stem cell-based strategies for cartilage repair and regeneration. Stem Cell Rev. Rep..

[31] Hofer H.R., Tuan R.S. (2016). Secreted trophic factors of mesenchymal stem cells support neurovascular and musculoskeletal therapies. Stem Cell Res..

[32] Tao S.C., Yuan T., Zhang Y.L., Yin W.J., Guo S.C., Zhang C.Q. (2017). Exosomes derived from miR-140-5p-overexpressing human synovial mesenchymal stem cells enhance cartilage tissue regeneration and prevent osteoarthritis of the knee in a rat model. Theranostics.

[33] Wang Y., Yu D., Liu Z., Zhou F., Dai J., Wu B., Zhou J., Heng B.C., Zou X.H., Ouyang H. (2017). Exosomes from embryonic mesenchymal stem cells alleviate osteoarthritis through balancing synthesis and degradation of cartilage extracellular matrix. Stem Cell Res..

[34] Zhu Y., Wang Y., Zhao B., Niu X., Hu B., Li Q., Zhang J., Ding J., Chen Y., Wang Y. (2017). Comparison of exosomes secreted by induced pluripotent stem cell-derived mesenchymal stem cells and synovial membrane-derived mesenchymal stem cells for the treatment of osteoarthritis. Stem Cell Res..

[35] Zhang S., Chu W.C., Lai R.C., Lim S.K., Hui J.H., Toh W.S. (2016). Exosomes derived from human embryonic mesenchymal stem cells promote osteochondral regeneration. Osteoarthr. Cartil..

[36] Mao Z., Li Y., Yang Y., Fang Z., Chen X., Wang Y., Kang J., Qu X., Yuan W., Dai K. (2018). Osteoinductivity and Antibacterial Properties of Strontium Ranelate-Loaded Poly(Lactic-co-Glycolic Acid) Microspheres With Assembled Silver and Hydroxyapatite Nanoparticles. Front. Pharm..

[37] Wu J., Kuang L., Chen C., Yang J., Zeng W.-N., Li T., Chen H., Huang S., Fu Z., Li J. (2019). miR-100-5p-abundant exosomes derived from infrapatellar fat pad MSCs protect articular cartilage and ameliorate gait abnormalities via inhibition of mTOR in osteoarthritis. Biomaterials.

[38] Zavatti M., Beretti F., Casciaro F., Bertucci E., Maraldi T. (2020). Comparison of the therapeutic effect of amniotic fluid stem cells and their exosomes on monoiodoacetate-induced animal model of osteoarthritis. Biofactors.

[39] Guillot P.V., Gotherstrom C., Chan J., Kurata H., Fisk N.M. (2007). Human first-trimester fetal MSC express pluripotency markers and grow faster and have longer telomeres than adult MSC. Stem Cells.

[40] Stenderup K., Justesen J., Clausen C., Kassem M. (2003). Aging is associated with decreased maximal life span and accelerated senescence of bone marrow stromal cells. Bone.

[41] Zhang Z.Y., Teoh S.H., Chong M.S., Schantz J.T., Fisk N.M., Choolani M.A., Chan J. (2009). Superior osteogenic capacity for bone tissue engineering of fetal compared with perinatal and adult mesenchymal stem cells. Stem Cells.

[42] Campagnoli C., Roberts I.A., Kumar S., Bennett P.R., Bellantuono I., Fisk N.M. (2001). Identification of mesenchymal stem/progenitor cells in human first-trimester fetal blood, liver, and bone marrow. Blood.

[43] Caplan A.I. (1994). The mesengenic process. Clin. Plast. Surg..

[44] Mattar P., Bieback K. (2015). Comparing the immunomodulatory properties of bone marrow, adipose tissue, and birth-associated tissue mesenchymal stromal cells. Front. Immunol..

[45] Ullah I., Subbarao R.B., Rho G.J. (2015). Human mesenchymal stem cells-current trends and future prospective. Biosci. Rep..

[46] Gotherstrom C., Ringden O., Westgren M., Tammik C., Le Blanc K. (2003). Immunomodulatory effects of human foetal liver-derived mesenchymal stem cells. Bone Marrow Transpl..

[47] Götherström C., Lundqvist A., Duprez I.R., Childs R., Berg L., le Blanc K.J.C. (2011). Fetal and adult multipotent mesenchymal stromal cells are killed by different pathways. Cytotherapy.

[48] Gotherstrom C., Ringden O., Tammik C., Zetterberg E., Westgren M., Le Blanc K. (2004). Immunologic properties of human fetal mesenchymal stem cells. Am. J. Obs. Gynecol..

[49] Kim M., Kim C., Choi Y.S., Kim M., Park C., Suh Y. (2012). Age-related alterations in mesenchymal stem cells related to shift in differentiation from osteogenic to adipogenic potential: Implication to age-associated bone diseases and defects. Mech. Ageing Dev..

[50] Guillot P.V., De Bari C., Dell’Accio F., Kurata H., Polak J., Fisk N.M. (2008). Comparative osteogenic transcription profiling of various fetal and adult mesenchymal stem cell sources. Differentiation.

[51] Kim S., Lee S.K., Kim H., Kim T.M. (2018). Exosomes Secreted from Induced Pluripotent Stem Cell-Derived Mesenchymal Stem Cells Accelerate Skin Cell Proliferation. Int. J. Mol. Sci..

[52] Zhang J., Liu X., Li H., Chen C., Hu B., Niu X., Li Q., Zhao B., Xie Z., Wang Y. (2016). Exosomes/tricalcium phosphate combination scaffolds can enhance bone regeneration by activating the PI3K/Akt signaling pathway. Stem Cell Res..

[53] Arslan F., Lai R.C., Smeets M.B., Akeroyd L., Choo A., Aguor E.N., Timmers L., van Rijen H.V., Doevendans P.A., Pasterkamp G. (2013). Mesenchymal stem cell-derived exosomes increase ATP levels, decrease oxidative stress and activate PI3K/Akt pathway to enhance myocardial viability and prevent adverse remodeling after myocardial ischemia/reperfusion injury. Stem Cell Res..

[54] Barberini D.J., Freitas N.P.P., Magnoni M.S., Maia L., Listoni A.J., Heckler M.C., Sudano M.J., Golim M.A., da Cruz Landim-Alvarenga F., Amorim R.M. (2014). Equine mesenchymal stem cells from bone marrow, adipose tissue and umbilical cord: Immunophenotypic characterization and differentiation potential. Stem Cell Res..

[55] Ranera B., Lyahyai J., Romero A., Vazquez F.J., Remacha A.R., Bernal M.L., Zaragoza P., Rodellar C., Martin-Burriel I. (2011). Immunophenotype and gene expression profiles of cell surface markers of mesenchymal stem cells derived from equine bone marrow and adipose tissue. Vet. Immunol. Immunopathol..

[56] Xie L., Zhang N., Marsano A., Vunjak-Novakovic G., Zhang Y., Lopez M.J. (2013). In vitro mesenchymal trilineage differentiation and extracellular matrix production by adipose and bone marrow derived adult equine multipotent stromal cells on a collagen scaffold. Stem Cell Rev. Rep..

[57] Hillmann A., Ahrberg A.B., Brehm W., Heller S., Josten C., Paebst F., Burk J. (2016). Comparative Characterization of Human and Equine Mesenchymal Stromal Cells: A Basis for Translational Studies in the Equine Model. Cell Transpl..

[58] Capomaccio S., Cappelli K., Bazzucchi C., Coletti M., Gialletti R., Moriconi F., Passamonti F., Pepe M., Petrini S., Mecocci S. (2019). Equine Adipose-Derived Mesenchymal Stromal Cells Release Extracellular Vesicles Enclosing Different Subsets of Small RNAs. Stem Cells Int..

[59] Pascucci L., Alessandri G., Dall’Aglio C., Mercati F., Coliolo P., Bazzucchi C., Dante S., Petrini S., Curina G., Ceccarelli P. (2014). Membrane vesicles mediate pro-angiogenic activity of equine adipose-derived mesenchymal stromal cells. Vet. J..

[60] Klymiuk M.C., Balz N., Elashry M.I., Heimann M., Wenisch S., Arnhold S. (2019). Exosomes isolation and identification from equine mesenchymal stem cells. BMC Vet. Res..

[61] Kojima M., Gimenes-Junior J.A., Langness S., Morishita K., Lavoie-Gagne O., Eliceiri B., Costantini T.W., Coimbra R. (2017). Exosomes, not protein or lipids, in mesenteric lymph activate inflammation: Unlocking the mystery of post-shock multiple organ failure. J. Trauma Acute Care Surg..

[62] Kowal J., Arras G., Colombo M., Jouve M., Morath J.P., Primdal-Bengtson B., Dingli F., Loew D., Tkach M., Thery C. (2016). Proteomic comparison defines novel markers to characterize heterogeneous populations of extracellular vesicle subtypes. Proc. Natl. Acad. Sci. USA.

[63] Xiao B., Chai Y., Lv S., Ye M., Wu M., Xie L., Fan Y., Zhu X., Gao Z. (2017). Endothelial cell-derived exosomes protect SH-SY5Y nerve cells against ischemia/reperfusion injury. Int. J. Mol. Med..

[64] Schey K.L., Luther J.M., Rose K.L. (2015). Proteomics characterization of exosome cargo. Methods.

[65] Kim J., Shin H., Kim J., Kim J., Park J. (2015). Isolation of High-Purity Extracellular Vesicles by Extracting Proteins Using Aqueous Two-Phase System. PLoS ONE.

[66] Shin H., Park Y.H., Kim Y.G., Lee J.Y., Park J. (2018). Aqueous two-phase system to isolate extracellular vesicles from urine for prostate cancer diagnosis. PLoS ONE.

[67] Skotland T., Sagini K., Sandvig K., Llorente A. (2020). An emerging focus on lipids in extracellular vesicles. Adv. Drug Deliv. Rev..

