# Supplementary material for: Nanoparticles from Equine Fetal Bone Marrow-Derived Cells Enhance the Survival of Injured Chondrocytes
Source: Animals (Basel). 2020 Sep 23;10(10):1723. doi: 10.3390/ani10101723 (PMC7598183; doi:10.3390/ani10101723)

Appendix A (Supplementary Materials and Methods)

Table S1. The primer sequences for qRT-PCR.

Gene	Forward (5′ → 3′)	Reverse (3′ → 5′)
Gapdh	TCCCTGCTTCTACTGGTGCT	CGTATTTGGCAGCTTTCTCC
TGF-β1	AGGCTCAAGTTAAGCGTGGA	CAGCCGGTTACTGAGGTAGC
IDO	CATTGTGATTCCTGCACACC	ACATCAGTGCCTCCAGTTCC
IL-10	CAAGCCTTGTCGGAGATGAT	CTCACTCGGAGGGTCTTCAG

Appendix B (Supplementary Data)

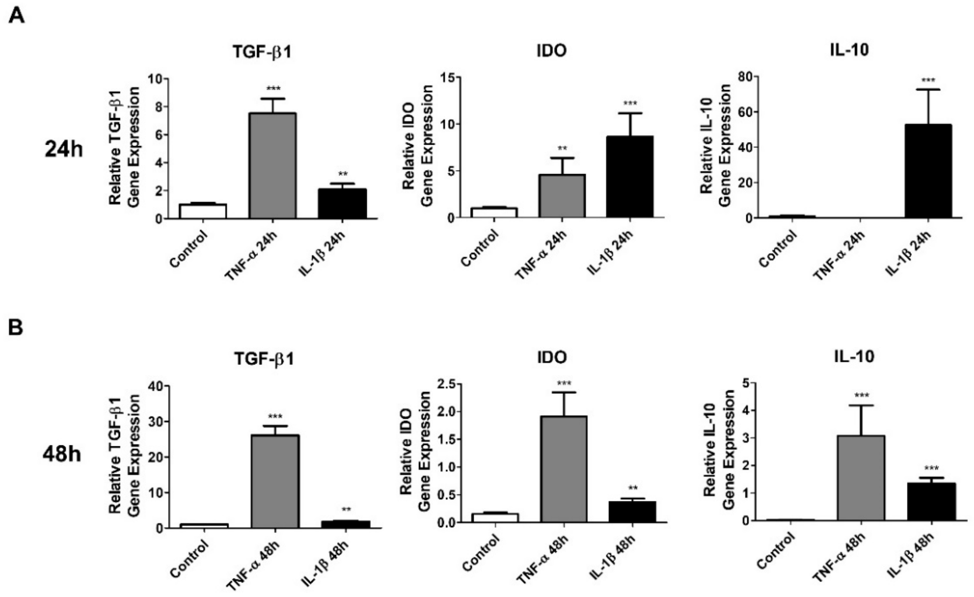

Figure S1. The mRNA expression analysis of immunoregulatory genes in BMCs. The expression of genes was examined after being treated with TNF-α and IL-1β for 24 h (**A**) and 48 h (**B**). * $p < 0.05$, ** $p < 0.01$, and *** $p < 0.005$ compared to untreated control.

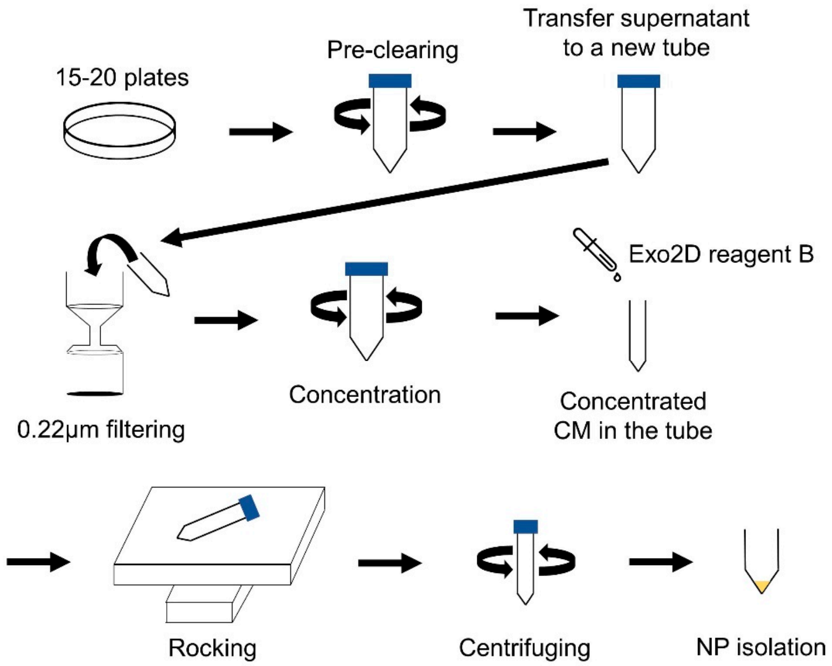

Figure S2. The schematic workflow of isolation of BMC-NPs.

Original images of Figure 2C

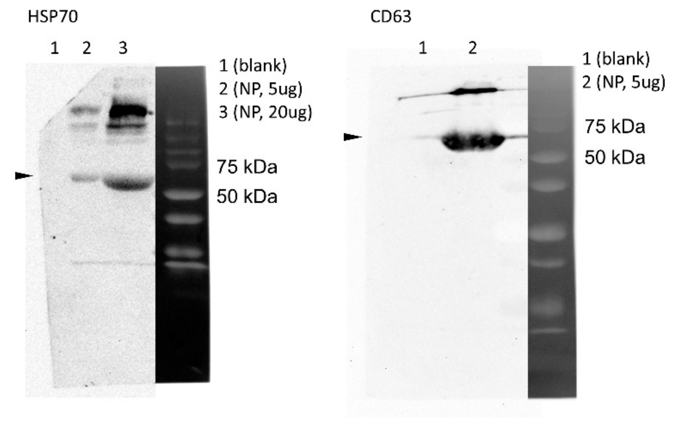

Supplement: Supplementary file 1 [file animals-10-01723-s001.pdf]
